# Personalized approach to growth hormone replacement in adults

**DOI:** 10.20945/2359-3997000000189

**Published:** 2019-11-01

**Authors:** Christa C. van Bunderen, Camilla Glad, Gudmundur Johannsson, Daniel S. Olsson

**Affiliations:** 1 Vrije Universiteit Amsterdam Department of Internal Medicine Sub-section of Endocrinology Amsterdam Netherlands Amsterdam UMC, Vrije Universiteit Amsterdam, Department of Internal Medicine, Sub-section of Endocrinology, Amsterdam, Netherlands; 2 Department of Internal Medicine and Clinical Nutrition Institute of Medicine at Sahlgrenska Academy University of Gothenburg Göteborg Sweden Department of Internal Medicine and Clinical Nutrition, Institute of Medicine at Sahlgrenska Academy, University of Gothenburg, Göteborg, Sweden,; Department of Endocrinology Sahlgrenska University Hospital Göteborg Sweden and Department of Endocrinology, Sahlgrenska University Hospital, Göteborg, Sweden

**Keywords:** Growth hormone, growth hormone replacement therapy, individualized medicine, tailoring of treatment

## Abstract

Growth hormone (GH) deficiency (GHD) in adults is well-characterized and includes abnormal body composition, reduced bone mass, an adverse cardiovascular risk profile, and impaired quality of life. In the early 1990s, it was also shown that patients with hypopituitarism without GH replacement therapy (GHRT) had excess mortality. Today, GHRT has been shown to decrease or reverse the negative effects of GHD. In addition, recent papers have shown that mortality and morbidity are approaching normal in hypopituitary patients with GHD who receive modern endocrine therapy including GHRT. Since the first dose-finding studies, it has been clear that efficacy and side effects differ substantially between patients. Many factors have been suggested as affecting responsiveness, such as sex, age, age at GHD onset, adherence, and GH receptor polymorphisms, with sex and sex steroid replacement having the greatest impact. Therefore, the individual tailoring of GH dose is of great importance to achieve sufficient efficacy without side effects. One group that stands out is women receiving oral estrogen replacement, who needs the highest dose. Serum insulin-like growth factor-1 (IGF-1) is still the most used biochemical biomarker for GH dose titration, although the best serum IGF-1 target is still debated. Patients with GHD due to acromegaly, Cushing’s disease, or craniopharyngioma experience similar effects from GHRT as others. Arch Endocrinol Metab. 2019;63(6):592-600

## INTRODUCTION

Growth hormone (GH) deficiency (GHD) has been a well-known clinical entity for a long time in children. To mitigate decreased linear growth in children, GH replacement therapy (GHRT) has been used since the late 1950s ( 1 ). Before 1985, when recombinant GH was approved in the US, the limited supply of pituitary-derived GH hindered research into its use in adults with GHD. Although a few early reports on the effects of GHRT in hypopituitary adults had already been published in the 1960s ( 2 ) and hypotheses had been made on the impact that GH might have in adults based on clinical studies in hypopituitarism ( 3 ), it was not until the availability of recombinant human GH that the research field of adult GHD was established.

Today, the consequences of GHD in adults are well defined and well described ( 4 , 5 ). GHD in adults is most commonly caused by a pituitary or peripituitary tumor or its treatments. The main features of adult GHD include abnormal body composition, reduced muscle strength and exercise capacity, reduced bone mass, an adverse cardiovascular risk profile, and impaired quality of life. The abnormal body composition includes increased fat mass with abdominal distribution, and reduced muscle mass and extracellular water content. GHD is also associated with an abnormal plasma lipid profile. In addition, patients with GHD and hypopituitarism have been shown to have excess cardiovascular morbidity and mortality ( 6 , 7 ).

Since the first two double-blind, placebo-controlled studies of GHRT in adults were reported in 1989, daily GHRT has been shown to reverse or ameliorate many of the symptoms associated with adult GHD ( 8 , 9 ). A meta-analysis of randomized, blinded, placebo-controlled trials showed that long-term GHRT has beneficial effects on muscle and fat mass, lipid profile, and diastolic blood pressure ( 10 ). Long-term GHRT has also been shown to increase bone mass and to improve quality of life, especially energy level and emotional reaction ( 11 , 12 ). Furthermore, recent papers have shown that morbidity and mortality are close to normal in hypopituitary patients with GHD receiving modern replacement therapy, including GHRT and if needed personalized substitution of other pituitary axes ( 13 , 14 ).

The aim of this review is to describe the individual variability in response to GHRT and, therefore, the importance of individualization of the treatment.

## INDIVIDUAL RESPONSE TO GHRT

The first studies reporting on the efficacy and safety of GHRT in adults used a weight-based dose regimen which was basically copied from pediatric practice ( 8 , 9 , 15 ). Side effects related to fluid retention induced by GH and the increased serum insulin-like growth factor-1 (IGF- 1 ) were common. It soon became clear that older patients and those with higher body weight had a higher frequency of side effects ( 16 ), suggesting that an individualized approach was needed in order to reduce the prevalence of side effects and, at the same time, obtain similar efficacy ( 17 - 19 ). This was the start of the use of the dose-titration regimen which is today recommended by clinical guidelines ( 20 , 21 ).

### Sex differences

Observations were made in some of the early studies that the response to GHRT differed markedly among patients ( 17 ). The most obvious difference was that observed between the sexes, with men showing a more marked effect on body composition with a higher increase in lean body mass and extracellular water, and larger reduction in fat mass in response to GHRT ( 22 , 23 ). Men also showed a more marked increase in serum IGF-1 in response to GHRT than women ( 17 ). This difference has been explained by the different interactions between GH and testosterone and estrogen, respectively. Testosterone augments the effects of GH through increasing muscle mass and extracellular water volume ( 24 ). Estrogen, on the other hand, may increase fat mass and reduce the serum IGF-1 response to GH. This effect of estrogen is dependent on the route of administration, as oral administration exposes the liver to supraphysiological estrogen doses in order to achieve physiological systemic exposure of estrogen due to first-pass hepatic metabolism ( 25 ). Oral estrogen, but not transdermal estrogen, therefore attenuates the increase in serum IGF-1 concentration in response to GH and reduces lipid oxidation ( 25 , 26 ). These effects help explain the sex differences seen in body composition in response to GHRT.

### Effect of age at GHD onset

In some of the earlier studies on adult GHD patients, it became clear that the outcome of therapy differed depending on the timing of GHD onset, i.e. childhood-onset or adult-onset of GHD. Childhood-onset patients had more severe consequences in terms of changes in body composition (increased body fat, decreased lean body mass and decreased bone mass) ( 27 ), whereas their self-perceived quality of life was better than among adult-onset patients ( 28 ). The improvement in quality of life was less pronounced among those with childhood-onset disease ( 28 ), whereas the initial response in body composition was more marked ( 27 ). In a prospective 5-year follow-up study comparing adult-onset and childhood-onset GHD, which was well matched for age, sex, and degree of hypopituitarism, the long-term effects on body composition, bone mineral density, and muscle strength were similar, although the initial response was more marked in patients with childhood-onset disease ( 27 ). A confounder in these comparisons is that childhood-onset patients usually have a longer duration of GHD.

Whether age *per se* has an impact on the response to GHRT is unclear. During logistic regression analysis in a prospective single-center study, age was not found to be a significant predictor of response in terms of serum IGF-1 and body composition ( 29 ). Another large post-marketing database analysis showed that older patients responded to GHRT similarly to younger patients in terms of waist circumference, blood pressure, and lipids ( 30 ). Taken together, these data suggest that age *per se* is not a major predictor of the response to GHRT among adults.

### Pharmacogenetics

While sex and age at onset of GHD explain some of the variability in the responsiveness to GHRT, other factors such as individual genotype may be of additional importance. To date, by far the most intensively studied genetic variant in this respect has been the GH receptor exon 3-deleted/full-length polymorphism. At a genetic level, each individual carries either two copies of the full-length receptor, two copies of the exon 3-deleted receptor (d 3 -receptor), or one of each (heterozygote). It was initially shown in a pediatric study that patients carrying the d3-receptor had a better growth response to GHRT than those with the full-length receptor ( 31 ). Since then, several studies in both children and adults have been performed in an attempt to further investigate the impact of the GH receptor isoforms on clinical response to GHRT and their findings have been detailed in a recent review ( 32 ). In summary, trials with a duration longer than 6 months showed that patients carrying the d3-receptor isoform had a marginally larger serum IGF-1 response, i.e. lower GH dose achieved the same IGF-1 response and, in some studies, a more favorable response in plasma lipids. On the other hand, one of the largest studies investigating the very rapid serum IGF-1 response after only a week of treatment showed a better serum IGF-1 response, after elimination of confounders (e.g. dose titration, adherence), in patients who were homozygous for the full-length receptor ( 33 ). Other studies, however, did not show any impact of GH receptor polymorphism on treatment response ( 32 ).

GH has various effects on humans, both directly and indirectly through IGF-1. For example, GH has important effects on hepatic lipoprotein metabolism that are mainly mediated through the GH receptor in the liver, and GH and IGF-1 increase extracellular water by affecting the renal tubules and through other mechanisms. Genotypes related to the functionality of these systems may therefore affect the responsiveness to GHRT. This concept has been explored in two different trials. In 318 adult patients with hypothalamic-pituitary disorder, the response to 1-year of GHRT was studied in terms of lipid metabolism ( 34 ). Serum total cholesterol and low-density lipoprotein-cholesterol concentrations decreased, and serum high-density lipoprotein-cholesterol concentration increased. Among 20 pre-selected single-nucleotide polymorphisms (SNPs), two SNPs were found to be associated with the response to GHRT. The APOB SNP rs676210 GG genotype was associated with larger reductions in total cholesterol and low-density lipoprotein-cholesterol, and the PPARG SNP rs10865710 CC genotype with greater reduction in total cholesterol. In another study, the association between 19 pre-selected SNPs in genes related to renal tubular function and the extracellular water response after 1-year of GHRT were investigated ( 35 ). None of the studied SNPs had a significant impact during GHRT.

In summary, one particular polymorphism in the GH receptor gene has been associated with treatment response in some studies. The association is, however, weak and of little clinical significance when trying to identify those patients that would benefit the most from GHRT. Using genotype related to the end-points of interest for GHRT has also been able to show some associations, in particular in relation to lipid metabolism, but again the effect size of these associations is too small to be of clinical relevance, although they indicate a functional relationship.

## ADHERENCE

For all long-term treatments, adherence is one of the factors that determines treatment success together with patient characteristics, therapy area challenges, treatment access, and healthcare provision ( 36 ). Individual responsiveness is therefore very likely influenced by adherence. Very few studies have examined the adherence of adult patients receiving long-term GHRT. One study examined adherence in 179 adult patients receiving GHRT assessed by calculating the percentage of available prescription data compared to recommended GH dosages over a mean follow-up period of approximately 7 years ( 37 ). Adherence during the first year was 85% and then gradually decreased, with the largest decline during the first 2 years. Young age and childhood onset of GHD were associated with low adherence, but not with the underlying diagnosis and the degree of hypopituitarism. In addition, no association was seen between change in serum IGF-1 concentration and adherence, suggesting that serum IGF-1 is not a reliable marker to assess individual adherence. Another study showed that approximately 35% of adult patients have poor adherence and were also skeptical of GHRT. This study also showed that younger age and childhood onset of GHD were associated with the poorest adherence ( 38 ).

Adherence is likely to be an important factor for individual responsiveness, although this has not been extensively studied. Factors that can be modified, based on pediatric studies, to improve adherence are lack of knowledge and understanding of the condition and its treatment, discomfort and pain associated with injections, and the quality of the healthcare professional-patient relationship ( 39 ).

## INDIVIDUAL TAILORING OF MANAGEMENT

### Dose titration

The weight- and body surface area-based dosing regimens of GHRT that were initially used were associated with adverse events (arthralgia and peripheral edema), especially in older, heavier, and female patients ( 16 ). Tailored dose-titration strategies with lower starting doses are therefore now recommended ( 19 - 21 ) ( Table 1 ). Total IGF-1 is still recognized as the most useful biochemical biomarker for GH dose titration in adults ( 40 ). Alongside individualized dosing, current clinical guidelines state that the goals of treatment should be an appropriate clinical response, avoidance of side effects, and serum IGF-1 value within the age-adjusted reference range ( 20 , 21 ). In clinical practice, it is still unclear how to dose GHRT and how to choose the appropriate target level for serum IGF-1 (within the age-adjusted reference ranges) in adults with GHD. Dose comparison studies have been conducted on the effect of low and high GH doses in relation to GHD at baseline ( 41 , 42 ). A beneficial effect of GHRT was demonstrated in both treatment arms with no significant difference between the groups. Serum IGF-1 was never used as a treatment target but, indirectly, no correlations were found between several outcome measures and serum IGF-1 level. A recent open-label, randomized clinical trial compared low-normal and high-normal IGF-1 target levels on various clinical endpoints during GHRT in adult GHD ( 43 , 44 ) ( Table 1 ). Increasing the GH dose in order to achieve IGF-1 levels between 1 and 2 standard deviation score (SDS) (adjusted for age and sex) improved waist circumference, microvascular function, and patient wellbeing. However, impaired effects on high-density lipoprotein-cholesterol in men, and insulin resistance and patient-reported myalgia in men and women were also seen with increasing the GH dose. In addition, when investigating cognitive function and mood in women, the adjustment of the GH dose seemed to have a narrow window. An increased dose appeared to impair prefrontal cognitive functioning, while a low dose resulted in decreased vigor. An IGF-1 target level between –1 and 1 SDS seems advisable; however, more scientific evidence is still warranted before implementation of this as a recommendation into current clinical practice. Gender, estrogen status, and age should be considered during dose titration due to the variability in response to GHRT. Furthermore, in certain special situations, an individualized choice of dose has to be made.

**Table 1:** Prospective studies investigating dose titration of GHRT

Authors	Year	No. of patients	Dose titration IGF-1 target level	Comparator	Duration (weeks)	Most important outcomes
van Bunderen et al. ( 44 )	2018	15	IGF-1 –2 to –1 SDS	IGF-1 –1 to 1 SDS	24	More patient-reported fatigue
		15	IGF-1 1 to 2 SDS			Decrease in waist circumference; decrease in HDL-cholesterol (men); more patient-reported myalgia
van Bunderen et al. ( 43 )	2016	15	IGF-1 –2 to –1 SDS	IGF-1 –1 to 1 SDS	24	Less vigor
		15	IGF-1 1 to 2 SDS			Deterioration of working memory and strategic memory
Drake et al. ( 18 )	1998	50 vs 21	IGF-1 0 to 2 SDS	Weight-based regimen	52	Rapid achievement of lower maintenance dose without loss of efficacy (QoL, waist circumference), with no sex difference
Johannsson et al. ( 19 )	1997	30 vs 30	IGF-1 –2 to 2 SDS + body composition	Weight-based regimen (12 mg/kg/day)	52	Fewer side effects; similar response to GHRT (body composition, glucose homeostasis, lipoprotein(a), blood pressure)

GHRT: growth hormone replacement therapy; HDL-cholesterol: high-density lipoprotein-cholesterol; IGF-1: insulin-like growth factor-1; QoL: quality of life; SDS: standard deviation score.

### Sex and age

Women are less responsive to GH, in particular those who are taking oral estrogens ( 25 , 26 ). Therefore, women have been shown to need a higher starting dose and need higher GH doses during long-term treatment to achieve the same IGF-1 target and clinical effect ( 17 , 20 , 21 , 45 ). This difference results in women being more likely to be undertreated.

In healthy subjects, IGF-1 declines with increasing age. Therefore, age should also be taken into account when choosing an appropriate IGF-1 target level ( 46 ). Age-adjusted reference values should be used to specify the target IGF-1 level. Initiating GHRT in elderly patients diagnosed with GHD is still debated. A systematic review by Kokshoorn and colleagues demonstrated clear improvements in lipid levels and quality of life in patients older than 60 years, but not in several other parameters ( 47 ). GHRT has similar efficacy in elderly hypopituitary patients with GHD, but at the same time these patients are more prone to develop side-effects to the treatment. At present there is no guidelines that recommend a chronological age when GHRT should be discontinued.

## SPECIAL SITUATIONS

### Cardiovascular disease and diabetes mellitus

Epidemiological studies show that hypopituitarism is associated with premature mortality due to cardiovascular and cerebrovascular disease. Beneficial effects of GHRT on cardiovascular risk factors are maintained for at least 10 years after initiation of therapy, with similar benefits seen in men and women when the GH dose is titrated to achieve a serum IGF-1 level between the median and the upper end of the age-related reference range ( 48 ). One theory as to why GHRT is beneficial with respect to cardiovascular disease is that a high-normal IGF-1 target level demonstrates a reduction of waist circumference, which contributes to improvement of vascular function through regulation of sympathetic nervous system activity in the microcirculation in a recent unpublished trial (unpublished data, C v Bunderen). However, the increased IGF-1 level with higher doses of GH has also demonstrated a negative impact on insulin resistance, which will deteriorate microcirculation ( 49 ). Data on the effect of GHRT in adults with GHD on glucose metabolism remains a matter of debate. The two registry studies in the field have shown an initially increased risk of diabetes mellitus during GHRT in one of the studies and no increased risk in the other ( 50 , 51 ). Epidemiological data on GHRT in non-functioning pituitary adenoma patients reported a normal incidence of type 2 diabetes mellitus ( 14 ). GH has an anti-insulin effect, but on the other hand, GHRT reduces abdominal fat mass and might therefore have a more beneficial effect on insulin resistance with long-term treatment ( 52 ). The increased insulin resistance obtained when increasing IGF-1 target level is likely to be due to a direct effect of increasing GH dose, with consequently increased lipolysis ( 53 ). Studies with very low GH dose (0.1 mg/day) demonstrated improved insulin sensitivity without affecting body composition ( 54 ). The authors of this paper postulated that this effect is mediated by the ability to increase IGF-1 bioavailability in the absence of the unfavorable lipolytic effects seen with higher doses of GH.

### Underlying diagnosis

### Acromegaly

Patients with GHD after previous acromegaly have an unfavorable metabolic profile which is comparable to that of patients with GHD after a non-functioning pituitary adenoma ( 55 ). GHRT in patients with previous acromegaly has been shown to improve metabolic parameters ( 56 ). However, contradictory results have also been published ( 57 ) and long-term data are sparse. The Dutch National Registry of long-term GHRT in adults demonstrated improvement of lipid profile in patients with previous acromegaly ( 58 ). However, glycated hemoglobin levels increased more compared to patients with non-functioning pituitary adenoma. GHRT in patients with GHD previously treated for acromegaly had no deleterious effect on cardiovascular morbidity. GHRT should not be withheld from patients with previous acromegaly, but close monitoring of glucose metabolism should be performed.

### Cushing’s disease

Patients with previous hypercortisolism show more long-term consequences of their disease, such as diabetes mellitus, hypertension, and fractures, compared to other etiologies of GHD ( 59 ). In multiple studies, patients with Cushing’s disease and patients with other etiologies responded similarly to GHRT, suggesting that patients with GHD due to Cushing’s disease benefit to the same extent as others ( 60 , 61 ).

### Pituitary tumor or malignancy

A study has raised some concern on the development of malignancies in children who received GHRT ( 62 ), but other studies are contradictory ( 63 ). A recent study on life expectancy in adults with pituitary adenoma receiving GHRT reported reduced overall mortality and a normal death rate due to cancer ( 13 ). Findings have also shown no association between cancer and serum IGF-1 level in adults with GHRT ( 64 ). Therefore, GHRT appears to be safe in GHD patients with respect to incidence and mortality due to malignancies ( 65 , 66 ). Patients with a craniopharyngioma have a marked excess all-cause mortality ( 67 ) and GHRT does not seem to resolve this increased mortality risk ( 65 , 68 ). GHRT together with other efforts related to the treatment of the underlying disorder and other pituitary hormone deficiencies should be further optimized in this patient population. Finally, long-term GHRT appears to be safe with regard to recurrence and enlargement of the primary pituitary tumor ( 69 , 70 ).

### Pregnancy

GHRT is not approved for use during conception and pregnancy. However, patients with hypopituitarism often conceive using assisted reproductive techniques and some studies support the benefit of GHRT in achieving fertility for women with GHD ( 71 ). The Pfizer International Metabolic Database (KIMS) analyzed pregnancies with different GHRT regimens, including stopping GHRT before pregnancy, as soon as pregnancy was confirmed, at the end of the second trimester, or continued throughout pregnancy. There was no relationship between the specific regimens and pregnancy outcome ( 72 ). Smaller studies have also demonstrated no negative influences of GHRT on maternal or fetal outcome ( 73 ). Physiologically, during gestation, the GH system is regulated by placental GH, which increases with the growth of the placenta and stimulates the maternal IGF-1 level, leading to a concomitant decline in the pituitary GH level ( 74 ). There is no consensus on how to manage GHRT during pregnancy, but no harm has been shown with either stopping GHRT before pregnancy, adjusting the dose, or continuing throughout the pregnancy.

## CONCLUSION

An individualized dose regimen with titration based on serum IGF-1 and clinical response should be used instead of weight- or body surface-based regimens to reduce side effects and to obtain similar efficacy. Women are less responsive to GH, i.e. the same GH dose results in a lower serum IGF-1 level and a lesser effect on body composition compared to men. This difference between the sexes is especially seen in women taking oral estrogens. In order to obtain similar efficacy in men and women, women will therefore need a longer dose titration period and a higher daily GH dose than men. In addition, age at GHD onset affects the responsiveness of GHRT in adults, e.g. adults with childhood onset of GHD seem to have a more marked initial response to GHRT in terms of changes in body composition even though the long-term effects seem to be similar. A patient’s genotype, including GH receptor isoforms, seems to have a weak effect on the responsiveness to GHRT and is, therefore, of little clinical significance. Even though not well investigated, adherence is most likely an important factor for responsiveness. Although the evidence is weak, it is suggested that individual GH dose is titrated on clinical response and towards a target serum IGF-1 level of 0 to 1 SDS without side effects.

Finally, the former excess mortality found in adult patients with hypopituitarism including GHD seems to approach normal with modern endocrine replacement therapy including GHRT. Furthermore, long-term GHRT appears to be safe regarding the incidence and mortality of malignancies, and the progression of the primary pituitary tumor. Many factors influence the response to GHRT, underlining the need for a personalized approach when treating patients with GHD.
